# Survivin promoter-regulated oncolytic adenovirus with Hsp70 gene exerts effective antitumor efficacy in gastric cancer immunotherapy

**DOI:** 10.18632/oncotarget.1430

**Published:** 2013-12-28

**Authors:** Weiguo Wang, Weidan Ji, Huanzhang Hu, Juming Ma, Xiaoya Li, Weiqun Mei, Yang Xu, Huizhen Hu, Yan Yan, Qizhe Song, Zhigang Li, Changqing Su

**Affiliations:** ^1^ Department of Internal Medicine, No. 117 Hospital of Chinese PLA, Hangzhou 310004, China.; ^2^ Department of Molecular Oncology, Eastern Hepatobiliary Surgical Hospital & National Center of Liver Cancer, Second Military Medical University, Shanghai 200438, China.; ^3^ Department of Hepatobiliary Surgery, Fuzhou General Hospital of Nanjing Military Area, Fuzhou 350025, China.; ^4^ Department of Cardiothoracic Surgery, Changhai Hospital, Second Military Medical University, Shanghai 200433, China.

**Keywords:** Gastric cancer, Survivin gene, Heat shock protein, Oncolytic adenovirus, Gene therapy

## Abstract

Gene therapy is a promising adjuvant therapeutic strategy for cancer treatment. To overcome the limitations of current gene therapy, such as poor transfection efficiency of vectors, low levels of transgene expression and lack of tumor targeting, the Survivin promoter was used to regulate the selective replication of oncolytic adenovirus in tumor cells, and the heat shock protein 70 (*Hsp70*) gene was loaded as the anticancer transgene to generate an AdSurp-Hsp70 viral therapy system. The efficacy of this targeted immunotherapy was examined in gastric cancer. The experiments showed that the oncolytic adenovirus can selectively replicate in and lyse the Survivin-positive gastric cancer cells, without significant toxicity to normal cells. AdSurp-Hsp70 reduced viability of cancer cells and inhibited tumor growth of gastric cancer xenografts in immuno-deficient and immuno-reconstruction mouse models. AdSurp-Hsp70 produced dual antitumor effects due to viral replication and high Hsp70 expression. This therapeutic system used the Survivin promoter-regulated oncolytic adenovirus vector to mediate targeted expression of the *Hsp70* gene and ensure safety and efficacy for subsequent gene therapy programs against a variety of cancers.

## INTRODUCTION

Gastric cancer is a great threat to human health, and gene therapy has become an important adjuvant therapeutic strategy for comprehensive gastric cancer treatment. The most frequently examined gene therapy approach in clinical trials includes adenovirus vectors [1, 2]. However, commonly used adenovirus vectors frequently fail to yield stable and persistent therapeutic effects due to various shortcomings, such as low gene transfection efficiency, poor targeting specificity and a short transgene expression. Thus, a key question in cancer gene therapy is how to design vectors in gene therapy systems that improve transfection efficiency, anticancer gene expression levels and tumor cell specificity.

Currently, the greatest challenge to cancer gene therapy is vector safety control. To improve adenovirus vector specificity and safety, researchers have generated multiple tumor-targeting replicative viral vectors that can lyse and kill various types of tumor cells by selectively replicating in such cells and releasing progeny viruses to infect additional tumor cells; these vectors are referred to as oncolytic viruses [3–5]. Our research group is committed to examining adenoviral vector techniques and has constructed oncolytic adenoviral vectors that use different targeting mechanisms [6–9]. Among such vectors, we produced a Survivin promoter-regulated oncolytic adenoviral vector with well-targeted oncolytic effects in both cell- and animal-model experiments for various cancers, including liver and gallbladder cancers [10, 11]. Survivin is frequently expressed by most human cancer tissues and has been closely correlated with elevated proliferative capacity, enhanced metastatic capacity, and chemotherapy and radiotherapy resistance in cancer cells. Thus, this Survivin promoter-regulated oncolytic adenoviral vector has broad-spectrum antitumor properties. We expect to use this vector in establishing broad-spectrum, specific, safe and effective anticancer gene therapy strategies.

Using the appropriate anticancer genes is important for improving gene therapy efficacy in cancer. In multiple studies on tumorigenesis and gene regulation, it was found that cells exposed to heat or other stress factors produce stress responses that involve inducing the heat shock protein (Hsp) expression; among such proteins, Hsp70 is closely related to immune regulation [12, 13]. Tumor cell-derived Hsp70 not only triggers specific immunity in tumor cells but are also potential molecular targets for NK (natural killer) cell recognition [14, 15]. Hsp70 plays an important role in antigen presentation and T cell proliferation [16]. Published studies have also demonstrated that Hsp70 is released in lysates after tumor cell necrosis and can promote dendritic cell (DC) maturation [17, 18]. Hsp70 can bind specific DC receptors and enhance host immune-response regulation by stimulating antigen-specific cytotoxic T cells and the Th1 response [19]. Thus, Hsp70 is an effective tumor inhibition factor and could, therefore, be a potent candidate gene for inducing antitumor immunity.

To improve the gene therapy approaches to tumor cell cytotoxicity, we used the aforementioned Survivin promoter-regulated oncolytic adenoviral vector to mediate Hsp70 gene expression, thereby producing targeted tumor cell-specific Hsp70 expression. This approach produced the synergistic effects, oncolytic adenoviral replication and anticancer gene-driven immunopotentiation, as well as improved safety and efficacy compared with prior antitumor gene therapy strategies. In particular, the approach developed herein overcomes the limitations of conventional gene therapy strategies, such as poor transfection efficiency, low transgene expression and weak tumor targeting. Moreover, the synergistic nature of the multiple antitumor mechanisms underlying this approach greatly enhanced the therapeutic effects of the proposed gene therapy technique.

## RESULTS

### Survivin expression in gastric cancer cells and its regulation of oncolytic adenovirus replication activity

**Figure 1:**
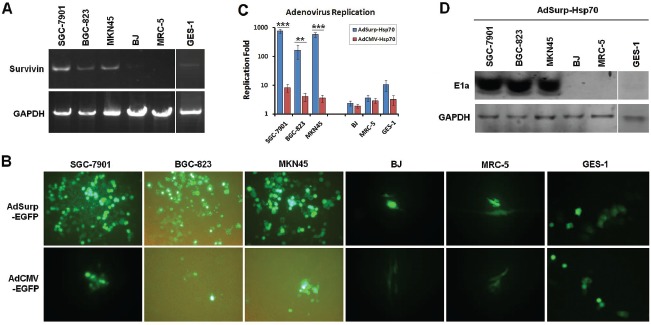
Survivin expression and oncolytic adenovirus replicative activity in cell lines examined (A) Cultured gastric cancer and normal cells were collected, and the TRIzol method was used to extract the total RNA from these cells. Survivin was amplified from the extracted RNA through RT-PCR with GAPDH as the internal control. (B) Fluorescence microscopy was used to observe EGFP-positive gastric cancer cells and normal cells 48 h after the cells were infected with AdSurp-EGFP or AdCMV-EGFP at an MOI of 1 pfu/cell, original magnification 200×. (C) Gastric cancer and normal cells were infected with AdSurp-Hsp70 or AdCMV-Hsp70 at an MOI of 1 pfu/cell. Forty-eight hours after infection, the cells were collected and the virus titer was quantified using the TCID_50_ assay. ** *P* < 0.01 and *** *P* < 0.001. (D) Gastric cancer and normal cells were infected with AdSurp-Hsp70 and AdCMV-Hsp70 at an MOI of 1 pfu/cell. Forty-eight hours after infection, the cells were collected, and Western blotting was used to detect E1a expression with GAPDH as the loading control.

RT-PCR analyses confirmed that Survivin was expressed in the SGC-7901, BGC-823 and MKN45 gastric cancer cell lines but not in the GES-1, BJ and MRC-5 normal cell lines (Fig. 1 A). The cancer cells exhibited significantly more fluorescence 48 h after they were infected with an adenovirus that carries the enhanced green fluorescent protein (EGFP) reporter gene (AdSurp-EGFP) at a multiplicity of infection (MOI) of 1 pfu/cell, compared with the initial stages of infection; however, no such increase in fuorescence was observed for normal cells. In contrast, fluorescence was not enhanced 48 h after AdCMV-EGFP infection in both cancer and normal cells (Fig. 1 B); thus, the Survivin promoter-regulated oncolytic virus AdSurp-EGFP exhibited specific replication activity in the gastric cancer cells.

Virus replication rates and E1a (early region 1a) expression levels were determined at 48 h after the cells were infected with AdSurp-Hsp70 or AdCMV-Hsp70 at an MOI of 10 pfu/cell. The results demonstrated that AdSurp-EGFP has significantly higher replication rates in the SGC-7901, BGC-823 and MKN45 gastric cancer cells than in the GES-1, BJ and MRC-5 normal cells (Fig. 1 C). In addition, after AdSurp-EGFP infection, the gastric cancer cells exhibited high E1a expression, whereas only weak E1a expression or negative expression was observed in normal cells (Fig. 1 D). However, AdCMV-Hsp70 exhibited no significant replication in either gastric cancer or normal cells, and E1a expression was completely negative in both cancer and normal cells.

### Oncolytic adenovirus-mediated Hsp70 expression specifically in gastric cancer cells

**Figure 2:**
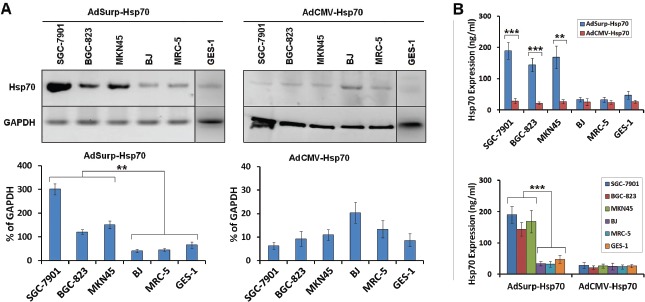
Survivin promoter-regulated oncolytic adenoviruses mediated Hsp70 expression (A) The cells were infected with the AdSurp-Hsp70 or AdCMV-Hsp70 adenovirus at a 10 pfu/cell MOI. Forty-eight hours after infection, the cells were collected, and western blotting was used to detect Hsp70 expression, with GAPDH as the loading control. The densitometry analysis was performed to show Hsp70 expression levels normalized with GAPDH density, ** *P* < 0.01. (B) The cells were infected with the AdSurp-Hsp70 or AdCMV-Hsp70 adenoviruses at a 10 pfu/cell MOI. Forty-eight hours after infection, the cells were collected, and an ELISA was used to detect Hsp70 expression. ** *P* < 0.01 and *** *P* < 0.001.

After infected with AdSurp-Hsp70 or AdCMV-Hsp70 at an MOI of 10 pfu/cell, cells and culture supernatant were collected 48 h after viral infection to determine Hsp70 expression levels through Western blotting and ELISA (enzyme-linked immunosorbent assay), respectively. The oncolytic virus AdSurp-Hsp70 mediated an elevated Hsp70 expression in gastric cancer cells, whereas lower Hsp70 levels were observed in normal cells; AdCMV-Hsp70 infection did not significantly increase Hsp70 expression in either gastric cancer or normal cells (Fig. 2 A–B).

### In vitro oncolytic adenovirus cytotoxicity in gastric cancer cells

**Figure 3:**
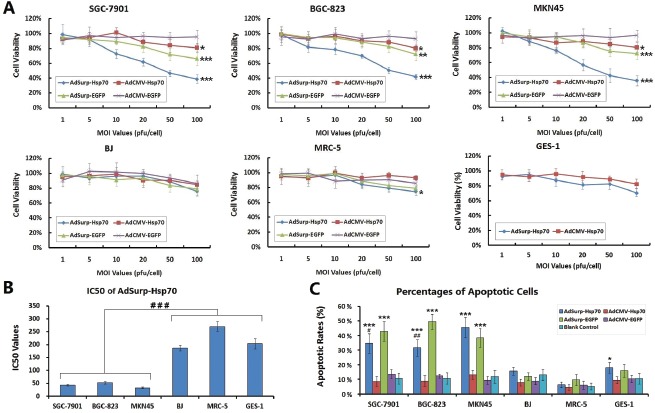
Specific cytotoxicity of oncolytic adenovirus in gastric cancer cells (A) The cells were seeded into 96-well plates at a concentration of 10^4^ cells/well and then infected with viruses at MOIs that ranged from 1 to 100 pfu/cell. Forty-eight hours after infection, cell viability was verified using the MTT assay, * *P* < 0.05, ** *P* < 0.01 and *** *P* < 0.001 compared with the control adenovirus AdCMV-EGFP. (B) The IC50 values that produced a 50% cell viability of viral MOI in the AdSurp-Hsp70 group were compared between cancer and normal cell lines, ^###^ *P* < 0.001. (C) Cells that were infected with viruses at an MOI of 50 pfu/cell were collected and examined the percentages of apoptotic cells by the terminal deoxynucleotidyl transferase-mediated dUTP nick end labeling (TUNEL) assay. The experiment was repeated three times, and the proportions of positive cells in each section were observed under 5 high power fields (×40 objective lens), * *P* < 0.05 and *** *P* < 0.001 compared with the blank control group in the same cell line; ^#^ *P* < 0.05 and ^##^ *P* < 0.01 compared with the AdSurp-EGFP group in the same cell line.

Gastric cancer and normal cells were infected with the AdSurp-Hsp70, AdCMV-Hsp70, AdSurp-EGFP or AdCMV-EGFP viruses at MOIs that ranged from 1 to 100 pfu/cell. Forty-eight hours after infection, cell viability was measured using the tetrazolium colorimetric examination cell proliferation (MTT) assay and the uninfected parental cells as controls. The results indicated that the gastric cancer cell viability gradually declined in the AdSurp-Hsp70, AdSurp-EGFP and AdCMV-Hsp70 infected cell groups as the MOI increased, but did not in the AdCMV-EGFP group. This antitumor effect was most evident in the AdSurp-Hsp70 group, wherein the cell viability decreased to 50% or less at an MOI of 50 pfu/cell, followed by the AdSurp-EGFP and AdCMV-Hsp70 groups. Thus, the replicative oncolytic adenovirus exhibited strong cytotoxic activity in gastric cancer cells. All the recombinant viruses produced similar effects in normal cells, cell viability slightly decreased at an MOI of 100 pfu/cell, but the cell viability remained at approximately 80% under such conditions (Fig. 3 A). We compared the MOI values that produced a 50% cell viability (IC50) in the AdSurp-Hsp70 group, which showed the IC50 values were 42.53±3.23 pfu/cell, 51.62±4.72 pfu/cell, 32.66±2.54 pfu/cell in SGC-7901, BGC-823, MKN45 cancer cells and 186.87±11.25 pfu/cell, 269.65±18.65 pfu/cell and 202.87±19.25 pfu/cell in BJ, MRC-5 and GES-1 cells, respectively, with significantly different IC50 values between the cancer cells and normal cells (Fig. 3 B).

The percentages of gastric cancer cell apoptosis were examined by the terminal deoxynucleotidyl transferase-mediated dUTP nick end labeling (TUNEL) assay. The oncolytic viruses AdSurp-Hsp70 and AdSurp-EGFP induced higher percentages of apoptosis in cancer cells than in normal cells, but the replication-defcient viruses AdCMV-Hsp70 and AdCMV-EGFP did not (Fig. 3 C). Additionally, the percentages of cell apoptosis were lower in the AdSurp-Hsp70-infected cancer cells than that in the AdSurp-EGFP-infected cancer cells, and this phenomenon appeared only in SGC-7901 and BGC-823 cells.

### Virus antitumor activity in the nude mouse xenografts

Two tumor models were established in BALB/C nude mice using SGC-7901 gastric cancer cells. In the first model (immuno-deficiency model), gastric cancer cells were subcutaneously implanted; after tumor formation, the mice were divided into groups, and viral therapy was then administered to the mice at totally 1×10^9^ pfu dosage of virus per mouse. In the second model (immuno-reconstitution model), the cytokine-induced killer (CIK) cells were injected into the mice after tumor formation to partially restore host immune function; the mice were then grouped and treated with the viruses using the procedures described for the first model.

**Figure 4:**
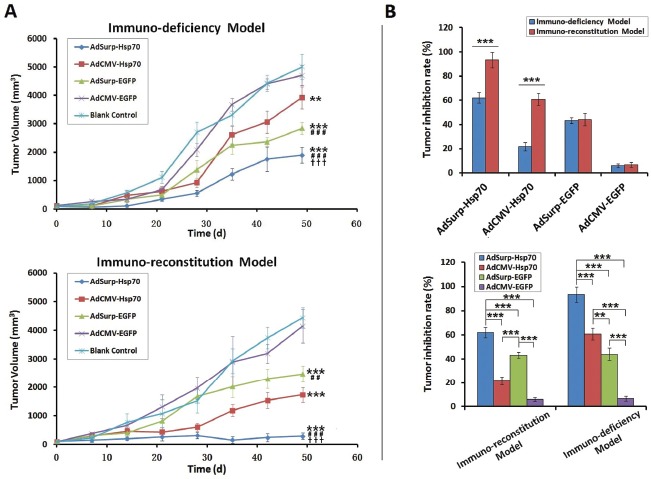
The efficacy of virus treatments in gastric cancer xenograft models (A) Nude mice were each implanted with 10^6^ SGC-7901 cells, which formed xenograft tumors 12 days thereafter. In the first immuno-defciency model, the mice were directly divided into treatment groups, and the mice in each group received 10^9^ pfu of the appropriate recombinant virus via multisite intratumoral injections. In the second immuno-reconstitution model, 10^7^ CIK cells were infused into each mouse before the mice were grouped and received the aforementioned virus therapies. The tumor diameters were measured weekly and used to calculate tumor volumes. ** *P* < 0.01 and *** *P* < 0.001 compared with the blank control group; ^##^ *P* < 0.01 and ^###^ *P* < 0.001 compared with the AdCMV-Hsp70 group; ^†††^ *P* < 0.001 compared with the AdSurp-Hsp70 group. (B) Data collected 49 days after the initial virus treatment were used to compare the tumor inhibition rates from viruses between these two models. ** *P* < 0.01 and *** *P* < 0.001.

In the first immuno-deficiency model, relative to the blank control mice, clear therapeutic effects were observed in the AdSurp-Hsp70 and AdSurp-EGFP treatment groups by 14 and 21 days, respectively, after the initial virus treatment (Fig. 4 A, upper panel). The observation period was terminated 49 days after virus treatment because the tumors in the control group mice exceeded the limitations established by the animal ethics committee. In the second immuno-reconstitution model, clear therapeutic effects were observed in the AdSurp-Hsp70 treatment group 7 days after the initial virus treatment and in the AdCMV-Hsp70 and AdSurp-EGFP treatment groups 14 days thereafter (Fig. 4 A, lower panel). The observation period was terminated 49 days after the initial viral treatment. We compared the virus treatment antitumor efficacy between these two models using the data of tumor inhibition rates collected 49 days after the initial virus treatment, which showed that the AdSurp-Hsp70 and AdCMV-Hsp70 virus antitumor effects were significantly greater in the immuno-reconstitution model than that in the immuno-deficiency one; in contrast, neither AdSurp-EGFP nor AdCMV-EGFP produced significantly different antitumor effects in the immuno-reconstitution model compared with the immuno-deficiency one (Fig. 4 B).

### Pathological observations on xenografted nude mouse tumor specimens

**Figure 5:**
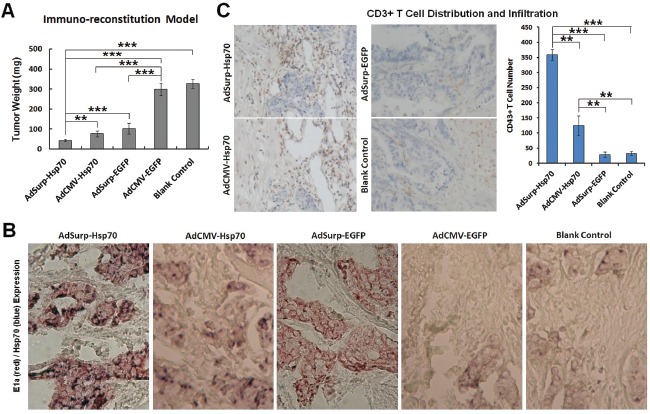
Pathological examinations on nude mouse xenograft specimens (A) The nude mouse xenograft tumor weights were compared. ** *P* < 0.01 and *** *P* < 0.001. (B) Immunohistochemical double staining to localize E1a (red) and Hsp70 (blue) expression in cancer cells, original magnification 200×. (C) Immunohistochemical staining to indicate the CD3+ T cell number and distribution that infiltrated the tumor stroma (DAB chromogen, yellowish brown), original magnification 200×. The number of positive cells was counted in 5 fields of view for each section using a 20× objective lens. ** *P* < 0.01 and *** *P* < 0.001.

At the end of the observation period for the immuno-reconstitution model, the mice were anesthetized through intraperitoneally injecting 3% sodium pentobarbital, and the tumor specimens were collected and weighed; the tumor weights were significantly lower in the AdSurp-Hsp70 group than in the other treatment groups (Fig. 5 A).

Tumor specimen paraffin sections were then subjected to immunohistochemical double staining to indicate expression of the adenovirus E1a gene and the target gene Hsp70. Sections from the AdSurp-Hsp70 and AdSurp-EGFP treatment groups exhibited positive E1a expression. The sections from these groups showed similar positive staining levels [3-amino-9-ethylcarbazole (AEC) chromogen; red indicated positive staining]; sections from the AdSurp-Hsp70 and AdCMV-Hsp70 treatment groups showed positive staining for Hsp70 with significantly stronger staining in sections from the AdSurp-Hsp70 group than in sections from the AdCMV-Hsp70 group [5-bromo-4-chloro-3-indolylphosphate (BCIP)/nitro blue tetrazolium (NBT) chromogen; blue indicated positive staining] (Fig. 5 B).

For the number of CD3+ T cells that infiltrated the tumor stroma, compared with the blank control group, we observed elevated T cell infiltration in the AdSurp-Hsp70 and AdCMV-Hsp70 groups with significantly greater infiltration in the AdSurp-Hsp70 group than the AdCMV-Hsp70 group (Fig. 5 C); the AdSurp-EGFP and AdCMV-EGFP groups did not significantly differ from the blank control group in number of infiltrating T cells.

## DISCUSSION

Currently, the greatest challenge facing gene therapy for cancer is vector safety. In 2011, results from successful cancer-related clinical trials were published in *Nature*. In these trials, an oncolytic viral vector with tumor-specific replicative activity was administered via intravenous infusion with successful vector targeting because the vector delivered the therapeutic genes to the cancer tissue [20, 21]. The trials validated oncolytic virus feasibility and superiority as gene therapy vectors. The conditionally replicating adenovirus could replicate in tumor cells and release progeny viruses to infect other tumor cells, thereby lysing and killing tumor cells; therefore, as discussed above, such viruses are known as oncolytic adenoviruses [22]. We constructed various tumor-specific promoter-regulated oncolytic adenoviruses wherein the early replicative genes Ela and Elb are controlled by a tumor-specific promoter. These viruses selectively replicated in cancer tissue without affecting normal tissue. Our therapeutic design is an important regulatory approach, which ensures that viruses specifically replicate in tumor cells. The fundamental basis for this approach includes rendering virus replication dependent on activation through a specific transcription factor in tumor cells. An upstream promoter, enhancer and other regulatory sequence may be used to specifically regulate a structural gene and activate targeted transcription expression of replicative genes. Oncolytic viral vectors specifically imported antitumor genes into tumor cells and effectively regulated their expression; thus, our approach killed tumor cells with reduced cytotoxic effects in normal cells. This approach contributes to gene therapy treatment program safety and effectiveness.

Many tissue- or cell-specific promoters have been identified and used to construct oncolytic viruses [7]. Among these candidate promoters, the Survivin promoter has attracted much attention due to its high specificity and applicability over a broad spectrum of tumors. Studies have demonstrated almost no Survivin expression in normal tissues but that Survivin is highly and selectively expressed in malignant tumors for most cancer types, including lung, liver, colon, pancreatic, prostate and breast cancers. Survivin expression is also closely associated with tumor recurrence and metastasis as well as a poor prognosis; thus, Survivin is a broad-spectrum molecular target for cancer gene therapy. In nude mouse xenograft experiments involving liver and gallbladder cancers, Survivin promoter-regulated oncolytic adenoviruses not only replicated in and lysed cancer cells in a targeted manner but also mediated high expression of the target antitumor genes [23–25]. Therefore, Survivin promoter-regulated oncolytic adenoviruses are expected to produce safe, broad-spectrum antitumor effects in most human tumors. In this study, we observed EGFP reporter gene expression and examined in vitro cytotoxicity to confirm that Survivin promoter-regulated oncolytic adenoviruses specifically replicated in three Survivin-positive gastric cancer cell lines and significantly decreased their viability without significant cytotoxicity to normal fibroblast cells.

Important considerations for cancer gene therapy applications include not only vector safety but also the role of antitumor gene selection for improved therapeutic efficiency. Our previous study demonstrated that stimulation by heat or other adverse stress stimulates cell stress responses that induce Hsp expression, which yields immunomodulatory effects [12]. Among the Hsp family proteins, Hsp70 plays an important role in immune responses, infection resistance and autoimmune reactions [26]. Tumor cell-derived Hsp70 induced specific immunity to tumor cells by activating NK cells and γ/δ T cells, which stimulated non-major histocompatibility complex (MHC)-restricted immune responses, activating the complement system and promoting multiple cytokine release with anti-tumor effects [19]. Therefore, Hsp70 is a robust antitumor candidate gene. However, the Hsp70 antitumor effects remain controversial, as certain studies have indicated that Hsp70 promotes tumor growth and downregulating Hsp70 expression may inhibit cancer cell proliferation [27].

In this study, we constructed the Survivin promoter-regulated oncolytic adenovirus AdSurp-Hsp70, which mediated high Hsp70 expression in tumor cells, and we used this virus for gastric cancer-targeted gene therapy. In vitro cytological experiments demonstrated that this vector replicated and expressed Hsp70 at high level in Survivin-positive gastric cancer cells, but it expressed Hsp70 at low level in the normal cell lines, which showed that this therapeutic strategy yields targeted gene expression. The adenovirus vectors AdSurp-Hsp70 and AdSurp-EGFP exhibited specific replicative activity in gastric cancer cells, lysed the cancer cells and induced cell apoptosis, thereby, significantly reduced the cancer cell survival rates. Relative to the blank control groups, AdCMV-Hsp70 did not replicate in gastric cancer cells and, therefore, did not yield oncolytic effects from viral infection. We also found that HSP70 prevents cell apoptosis in gastric cancer cell lines, SGC-7901 and BGC-823, when Hsp70 expression and cell apoptosis were at high levels mediated by AdSurp-Hsp70, but this pretective effect was not large enough to offset the apoptosis induced by viral oncolysis. Although the Hsp70-related immunomodulatory effect cannot be observed in the in vitro experiments herein, the expression of Hsp70 somewhat inhibited the gastric cancer cells, and its molecular mechanism is not clear. Hsp70 serves a critical survival function to prevent cancer cell apoptosis [26]. On the other hand, Hsp70 also can inhibit cancer cells by inducing the adaptive or innate immune response. The intracellular or extracellular location of Hsp70 is attributed to its dual activities that determine cancer cell to survival or death [28]. To enhance the inhibitory effect on cancer cells, we fused a membrane transduction peptide of 11 arginines (11R) into Hsp70, which was reported to be one of the most effective protein transduction domains (PTD) for introducing proteins into the cell membrane [29], thus, the modified transmembrane localization of Hsp70 may inhibit cancer cell growth in vitro and in vivo by regulating the transduction of growth factor signal pathways inside and outside of cells.

To further clarify the Hsp70 antitumor effects, we established two gastric cancer xenograft models in nude mice. In the first immuno-defciency model, SGC-7901 cells were implanted and grown in immunodefcient nude mice; the mice then received the virus treatments. In the second immuno-reconstitution model, SGC-7901 cells were implanted and grown in immunodefcient nude mice, but the mice then received CIK cell infusion to partially restore host immune function before receiving the virus treatments. Our results suggested that, in these two gastric cancer xenograft models, AdSurp-Hsp70 mediated high Hsp70 expression in cancer cells, which produced the dual tumor-inhibiting effects oncolytic virus propagation and Hsp70 expression in cancer cells. Thus, relative to the other examined viruses, this virus more reliably inhibited the xenograft tumor. In the immuno-reconstitution model, Hsp70 expression induced CD3+ T cell infiltration into tumor stroma, which suggested that high Hsp70 expression levels can strengthen a host's antitumor immune system responses.

In summary, we used a Survivin promoter-regulated oncolytic adenovirus and Hsp70 as an anticancer gene and generated results that will help ensure cancer gene therapy safety and effcacy. The experiments herein confirmed that in Survivin-positive gastric cancer cells, a Survivin promoter-regulated oncolytic adenovirus yields high replicative activity and mediates anticancer gene expression. Due to such effects, this adenovirus exhibited enhanced cytotoxicity in cancer cells relative to the additional viruses examined but did not greatly affect normal cells. This treatment approach may specifically target gastric cancer or other cancers to activate multiple anti-tumor mechanisms; thus, it overcomes the limitations of conventional gene therapy vectors, such as poor transfection rates, low gene expression levels and weak cancer cell targeting. Thus, this approach greatly improves antitumor effects relative to conventional gene therapy methods with prospects for broad development in widespread clinical applications.

## MATERIALS AND METHODS

### Cell lines and cell culture

The human gastric cancer cell lines SGC-7901, BGC-823 and MKN45, as well as the normal human fibroblast cell lines BJ and MRC-5, were purchased from the American Type Culture Collection (ATCC). The human embryonic kidney cell line HEK293 was purchased from the Microbix Biosystems (Ontario, Canada). The human gastric mucosa epithelial cell line GES-1 was kindly gifted by the Nanjing First Hospital, Nanjing Medical University (Nanjing, China) [30, 31]. The cells were cultured at 37°C and 5% CO_2_ in Dulbecco's modified Eagle's medium (DMEM) with 10% fetal bovine serum (FBS), 10 U/mL penicillin and 10 U/mL streptomycin.

### Survivin expression in the cell lines

The aforementioned cell lines were cultured in 6-well plates and collected during the logarithmic growth phase. The total RNA was extracted from the cells using the TRIzol reagent kit (Invitrogen Life Technologies, Carlsbad, CA, USA) in accordance with the manufacturer's instructions. Survivin expression was detected through reverse transcription polymerase chain reaction (RT-PCR) using the upstream primer 5'-cgg aat tca cca tgg gtg ccc cga cg-3' and downstream primer 5'-gaa gat ctt caa tcc atg gca gcc ag-3'. Survivin coding DNA (cDNA) was synthesized using the SuperScript^TM^ One-Step RT-PCR with Platinum® Taq Kit (Invitrogen Life Technologies) in accordance with the manufacturer's instructions. The following amplification conditions were used: 50°C for 30 min; 30 cycles of 94°C for 2 min, 94°C for 30 s, 60°C for 30 s and 72°C for 60 s; and 72°C for 2 min. The amplified fragment was 450 bp. The glyceraldehyde-3-phosphate dehydrogenase (GAPDH) was used as a control and was amplified using the upstream primer 5-acc aca gtc cat gcc atc ac-3' and downstream primer 5'-tcc acc acc ctg ttg ctt gta-3'.

### Adenoviral vector construction

The adenoviral vector used herein was created using the previously generated Survivin promoter-regulated oncolytic adenoviruses AdSurp-EGFP [10] and AdCMV-Hsp70 [7]. Full-length Hsp70 cDNA, which contains a sequence (CGCCGCAGGAGACGACGG CGACGGCGAAGAAGG) encoding a membrane transduction peptide of 11 arginines (11R) after the initiation codon, was amplified through PCR from AdCMV-Hsp70 using the upstream primer 5'-CCCAAGCTT ATGCGCCGCAGGAGACGACG-3', which included a Hind III cleavage site, and the downstream primer 5'-GCGTCGAC CTAATC TACCTCCTCAATGGTGGG-3', which included a Sal I cleavage site. The fragment released by Hind III and Sal I was sequenced, then inserted into the AdSurp-EGFP vector and replaced the EGFP gene to create the new adenovirus AdSurp-Hsp70. AdSurp-Hsp70 and the control adenoviruses AdSurp-EGFP, AdCMV-Hsp70 and AdCMV-EGFP [7] were amplified in HEK293 cells. The adenoviral DNA was extracted using the QIAamp DNA Blood Mini Kit (Qiagen, Inc., Valencia, CA) in accordance with the manufacturer's instructions. E1 region upstream and downstream primers (sense primer: 5'-GTG TAT TTA TAC CCG GTG AG-3'; antisense primer: 5'-TGG AAG ATT ATC AGC CAG TAC-3'), as well as Hsp70 upstream and downstream primers, were used to verify the viral sequence. The recombinant adenoviruses were then purified using conventional cesium chloride gradient centrifugation, and the TCID_50_ (50% tissue culture infective dose) assay (Qbiogene, Inc., IIIkich, France) was used to determine the virus titer.

### Oncolytic adenovirus replicative activity

Cells were plated into 6-well plates at 10^6^ cells/well and cultured for 24 h. Subsequently, the culture medium was replaced with serum-free medium, and AdSurp-EGFP or AdCMV-EGFP was added to the system at an MOI of 1 pfu/cell. After 2 h, the medium was discarded, medium with 5% serum was added, and the cells were cultured for an additional 48 h. The cells were then viewed using a fluorescence microscope to determine the EGFP-positive cell proportion and intensity.

Cells were plated into 6-well plates at 10^6^ cells/well and cultured for 24 h. Subsequently, the culture medium was replaced with a serum-free medium, and AdSurp-Hsp70 or AdCMV-Hsp70 was added to the system at an MOI of 1 pfu/cell. After 2 h, the medium was discarded, medium containing 5% serum was added, and the cells were cultured for an additional 48 h. The cells were then collected and subjected to three cycles of freezing at -80°C followed by thawing. A portion of the cell lysate was centrifuged, the precipitate was discarded, and the virus titer was determined for the supernatant using the TCID_50_ assay. An additional cell lysate sample was analyzed through Western blotting to detect expression for the adenovirus E1a gene. This blotting procedure used a monoclonal anti-adenovirus E1a antibody (Cell Signaling Technology, Inc., Shanghai, China) at the working concentration 1:500 followed by a horseradish peroxidase (HRP)-conjugated goat antimouse IgG (immunoglobulin G) antibody (Cell Signaling Technology, Inc.) at the working concentration 1:2000. Finally, a chromogenic solution with LumiGLO® chemiluminescent reagent and peroxide (Cell Signaling Technology, Inc.) was used to visualize the blots, which were then exposed to X-ray film for imaging.

### Oncolytic adenovirus-mediated Hsp70 gene expression

The cells were plated into 12-well plates at 10^5^/well and cultured for 24 h. The cells were then infected with AdSurp-Hsp70 or AdCMV-Hsp70 at an MOI of 10 pfu/cell using the procedures described above. After the infected cells were cultured for 48 h, the cells and culture supernatant were collected. The collected cells were subjected to three cycles of freezing at -80°C and thawing; the resulting cell lysate was centrifuged, and the supernatant was collected to detect Hsp70 protein expression levels through Western blotting. For Western blotting, we used 10% polyacrylamide gels, a monoclonal mouse anti-human Hsp70 antibody (Santa Cruz Biotechnology, Inc., Santa Cruz, CA, USA) at the working concentration 1:1000, HRP-conjugated goat antimouse IgG antibody (Cell Signaling Technology, Inc.) at the working concentration 1:2000 and a chromogenic solution with LumiGLO® chemiluminescent reagent and peroxide (Cell Signaling Technology, Inc.). The Hsp70 ELISA (Enzyme-linked immunosorbent assay) Kit (Stressgen Biotechnologies Corp., Victoria, Canada) was used to detect Hsp70 protein expression in the cell culture supernatant in accordance with the manufacturer's instructions.

### Specific cytotoxicity of the oncolytic adenovirus in cancer cells

The cytotoxic effects of viruses on gastric cancer cells in vitro was examined using MTT assay. Cells in the logarithmic growth phase were collected, seeded in 96-well plates at 10^4^ cells/well and cultured for 24 h. The culture medium was then replaced with a serum-free medium, and AdSurp-Hsp70, AdCMV-Hsp70, AdSurp-EGFP or AdCMV-EGFP was added at the MOI values of 1, 5, 10, 20, 50 or 100 pfu/cell; eight wells were used for each MOI value. After 2 h, the culture medium was replaced with medium that contained 5% serum. After culturing for 3 days, the culture medium was discarded

and replaced with 100 µl/well of 0.1 mol/L PBS and 10 µl/well of the MTT labeling reagent (Roche Diagnostics GmbH, Mannheim, Germany), which reached 0.5 mg/ml for the final concentration. The cells were incubated for 4 h; subsequently, 100 µl/well of solubilization solution [10% sodium dodecyl sulfate (SDS) in 0.01 mol/L HCl] was added, and the cells were incubated overnight. A Model 550 Microplate Reader (Model 550, BIO-RAD Laboratories, Tokyo, Japan) was then used to measure absorbance at 570 nm with the reference wavelength 655 nm, and cell survival curves were plotted.

Cells that were infected with AdSurp-Hsp70, AdCMV-Hsp70, AdSurp-EGFP or AdCMV-EGFP at an MOI of 50 pfu/cell were collected and examined the percentages of apoptotic cells by TUNEL staining (Fuzhou Maixin Biotechnology Development Co., Fuzhou, China) according to the provider's instructions. The experiment was repeated three times, and the proportions of positive cells in each section were observed under 5 high power fields (×40 objective lens).

### Antitumor experiments using a nude mice xenograft model

Fifty healthy 4-week-old purebred BALB/C nude mice were purchased from the Shanghai SLAC Laboratory Animal Center of the Chinese Academy of Sciences (Shanghai,China).

Twenty-five of these nude mice were subcutaneously injected on the right side of axilla with SGC-7901 gastric cancer cells; 10^6^ cells were injected per mouse. Twelve days after the injection, an approximate 0.5-cm diameter tumor appeared in each mouse. The mice were randomly divided into 5 groups (the AdSurp-Hsp70, AdCMV-Hsp70, AdSurp-EGFP, AdCMV-Hsp70 and blank control groups) with 5 mice in each group. In each group, the mice received direct multisite intratumor injections of the corresponding recombinant virus at 2×10^8^ pfu/mouse in 100 µl, once every other day, through 5 injections. Mice in the blank control group were injected 5 times with 100 µl of the viral storage solution (10 mmol/L Tris-HCl, pH 8.0; 2 mmol/L MgCl_2_; and 4% sucrose) instead of a recombinant adenovirus. Tumor growth was monitored regularly using vernier calipers to measure the tumor size, and the tumor volumes were calculated using the formula “*a*×*b*^2^×0.5” (*a* represents the maximum diameter, and *b* represents the minimum diameter). Tumor growth curves were then plotted using these measurements.

The remaining 25 nude mice were also injected with SGC-7901 cells using the above procedure. Tumors with an approximate 0.5-cm diameter appeared in these mice 12 days after the cancer cell injection. The mice then received pre-cultured CIK cell infusion by caudal vein injections; 10^7^ cells were injected per mouse. The CIK cells were derived from normal BALB/C mice using previously described cell collection, cell culture and in vitro stimulation methods [32]. The mice were then grouped for treatment and measurement as described above. At the end of the observation period, the mice were anesthetized through a 3% sodium pentobarbital intraperitoneal injection, and the tumor specimens were collected, weighed, fixed in 10% neutral buffered formalin and sliced into paraffin-embedded sections. Immunohistochemical double staining was used to determine the adenovirus E1a localization [using a monoclonal mouse anti-E1a antibody (Santa Cruz Biotechnology, Inc.) at the working concentration 1:200] and Hsp70 expression (with an antibody at the working concentration 1:100). In addition, a rabbit anti-mouse CD3+ antibody was used to label and count the CD3+ T cell number and distribution that infiltrated the tumor stroma. The number of positive cells was counted in 5 fields of view for each section using a 20× objective lens.

### Statistical analysis

The experimental data were expressed through “means±standard deviation”. Paired sample *t*-tests were used for pairwise data comparisons, whereas an analysis of variance (ANOVA) was used to compare multiple groups. The software PASW Statistics 18.0 was used for these analyses, and *P* < 0.05 was the statistical significance threshold.

## References

[1] Huang S, Kamihira M (2013). Development of hybrid viral vectors for gene therapy. Biotechnol Adv.

[2] Kron MW, Kreppel F (2012). Adenovirus vectors and subviral particles for protein and peptide delivery. Curr Gene Ther.

[3] Vacchelli E, Eggermont A, Sautès-Fridman C, Galon J, Zitvogel L, Kroemer G, Galluzzi L (2013). Trial watch: Oncolytic viruses for cancer therapy. Oncoimmunology.

[4] Meshii N, Takahashi G, Okunaga S, Hamada M, Iwai S, Takasu A, Ogawa Y, Yura Y (2013). Enhancement of systemic tumor immunity for squamous cell carcinoma cells by anoncolytic herpes simplex virus. Cancer Gene Ther.

[5] Li JM, Kao KC, Li LF, Yang TM, Wu CP, Horng YM, Jia WW, Yang CT (2013). MicroRNA-145 regulates oncolytic herpes simplex virus-1 for selective killing of human non-small cell lung cancer cells. Virol J.

[6] Xu C, Li H, Su C, Li Z (2013). Viral therapy for pancreatic cancer: tackle the bad guys with poison. Cancer Lett.

[7] Xu C, Sun Y, Wang Y, Yan Y, Shi Z, Chen L, Lin H, Lü S, Zhu M, Su C, Li Z (2012). CEA promoter-regulated oncolytic adenovirus-mediated Hsp70 expression in immune gene therapy for pancreatic cancer. Cancer Lett.

[8] Ma J, He X, Wang W, Huang Y, Chen L, Cong W, Gu J, Hu H, Shi J, Li L, Su C (2009). E2F promoter-regulated oncolytic adenovirus with p16 gene induces cell apoptosis and exerts antitumor effect on gastric cancer. Dig Dis Sci.

[9] Su C, Na M, Chen J, Wang X, Liu Y, Wang W, Zhang Q, Li L, Long J, Liu X, Wu M, Fan X, Qian Q (2008). Gene-viral cancer therapy using dual-regulated oncolytic adenovirus with antiangiogenesis gene for increased efficacy. Mol Cancer Res.

[10] Zhang Y, Fang L, Zhang Q, Zheng Q, Tong J, Fu X, Jiang X, Su C, Zheng J (2013). An oncolytic adenovirus regulated by a radiation-inducible promoter selectively mediates hSulf-1 gene expression and mutually reinforces antitumor activity of I131-metuximab in hepatocellular carcinoma. Mol Oncol.

[11] Liu C, Sun B, An N, Tan W, Cao L, Luo X, Yu Y, Feng F, Li B, Wu M, Su C, Jiang X (2011). Inhibitory effect of Survivin promoter-regulated oncolytic adenovirus carrying P53 gene against gallbladder cancer. Mol Oncol.

[12] Calderwood SK, Stevenson MA, Murshid A (2012). Heat shock proteins, autoimmunity, and cancer treatment. Autoimmune Dis.

[13] Chen T, Cao X (2010). Stress for maintaining memory: HSP70 as a mobile messenger for innate and adaptive immunity. Eur J Immunol.

[14] Böttger E, Multhoff G, Kun JF, Esen M (2012). Plasmodium falciparum-infected erythrocytes induce granzyme B by NK cells through expression of host-Hsp70. PLoS One.

[15] Chen X, Tao Q, Yu H, Zhang L, Cao X (2002). Tumor cell membrane-bound heat shock protein 70 elicits antitumor immunity. Immunol Lett.

[16] Chen T, Guo J, Han C, Yang M, Cao X (2009). Heat shock protein 70, released from heat-stressed tumor cells, initiates antitumor immunity by inducing tumor cell chemokine production and activating dendritic cells via TLR4 pathway. J Immunol.

[17] Srivastava P (2002). Interaction of heat shock proteins with peptides and antigen presenting cells: chaperoning of the innate and adaptive immune responses. Annu Rev Immunol.

[18] Morse MA, Clay TM, Hobeika AC, Osada T, Khan S, Chui S, Niedzwiecki D, Panicali D, Schlom J, Lyerly HK (2005). Phase I study of immunization with dendritic cells modified with fowlpox encoding carcinoembryonic antigen and costimulatory molecules. Clin Cancer Res.

[19] Kim TW, Lee JH, He L, Boyd DA, Hung CF, Wu TC (2005). DNA vaccines employing intracellular targeting strategies and a strategy to prolong dendritic cell life generate a higher number of CD8+ memory T cells and better long-term antitumor effects compared with a DNA prime-vaccinia boost regimen. Hum Gene Ther.

[20] Breitbach CJ, Burke J, Jonker D, Stephenson J, Haas AR, Chow LQ, Nieva J, Hwang TH, Moon A, Patt R, Pelusio A, Le Boeuf F, Burns J, Evgin L, De Silva N, Cvancic S, Robertson T, Je JE, Lee YS, Parato K, Diallo JS, Fenster A, Daneshmand M, Bell JC, Kirn DH (2011). Intravenous delivery of a multi-mechanistic cancer-targeted oncolytic poxvirus in humans. Nature.

[21] Galanis E (2011). Cancer: Tumour-fghting virus homes in. Nature.

[22] Patel MR, Kratzke RA (2013). Oncolytic virus therapy for cancer: the frst wave of translational clinical trials. Transl Res.

[23] Groner B, Weiss A (2013). Targeting Survivin in Cancer: Novel Drug Development Approaches. BioDrugs.

[24] Cao L, Li C, Shen S, Yan Y, Ji W, Wang J, Qian H, Jiang X, Li Z, Wu M, Zhang Y, Su C (2013). OCT4 increases BIRC5 and CCND1 expression and promotes cancer progression in hepatocellular carcinoma. BMC Cancer.

[25] Li C, Yan Y, Ji W, Bao L, Qian H, Chen L, Wu M, Chen H, Li Z, Su C (2012). OCT4 positively regulates Survivin expression to promote cancer cell proliferation and leads to poor prognosis in esophageal squamous cell carcinoma. PLoS One.

[26] Murphy ME (2013). The HSP70 family and cancer. Carcinogenesis.

[27] Behnsawy HM, Miyake H, Kusuda Y, Fujisawa M (2013). Small interfering RNA targeting heat shock protein 70 enhances chemosensitivity in human bladder cancer cells. Urol Oncol.

[28] Schmitt E, Gehrmann M, Brunet M, Multhoff G, Garrido C (2007). Intracellular and extracellular functions of heat shock proteins: repercussions in cancer therapy. J Leukoc Biol.

[29] Ogawa T, Ono S, Ichikawa T, Arimitsu S, Onoda K, Tokunaga K, Sugiu K, Tomizawa K, Matsui H, Date I (2009). Protein transduction method for cerebrovascular disorders. Acta Med Okayama.

[30] Wu HL, Duan ZT, Jiang ZD, Cao WJ, Wang ZB, Hu KW, Gao X, Wang SK, He BS, Zhang ZY, Xie HG (2013). Increased endoplasmic reticulum stress response is involved in clopidogrel-induced apoptosis of gastric epithelial cells. PLoS One.

[31] Pan Y, He B, Lirong Z, Nie Z, Chen L, Gu L, Hoffman AR, Wang S, Hu J (2013). Gene therapy for cancer through adenovirus vector-mediated expression of the Ad5 early region gene 1A based on loss of IGF2 imprinting. Oncol Rep.

[32] Todorovic M, Mesiano G, Gammaitoni L, Leuci V, Giraudo Diego L, Cammarata C, Jordaney N, Carnevale-Schianca F, Gallo S, Fagioli F, Piacibello W, Elia AR, Pignochino Y, Dell'aglio C, Grignani G, Cignetti A, Aglietta M, Sangiolo D (2012). Ex vivo allogeneic stimulation signifcantly improves expansion of cytokine-induced killer cells without increasing their alloreactivity across HLA barriers. J Immunother.

